# Consumption Cured by Subcutaneous Injections

**Published:** 1891-01

**Authors:** 


### CONSUMPTION CURED BY SUBCUTANEOUS INJECTIONS.

The medical world has become greatly agitated over the remarkable discovery by Professor Koch, of Berlin, of a fluid mixture—the ingredients of which are kept secret for the present—which, it is claimed when injected under the skin of a consumptive patient, successfully combats the disease and effects a permanent cure.

Professor Koch is a physician who has already acquired great distinction in his profession as the originator of the germ theory of disease, to the investigation of which he devoted a number of years, with results of inestimable value to mankind, and strange to say, the medical profession, usually so tardy to give place to any new thing, have adopted the Professor's views with great unanimity. His more recent discovery of a means of counteracting the ravages of the health destroying bacilli, has awakened a degree of interest in medical circles, which has not been equalled since the discoveries of the world renowned Pasteur. Like a true devotee of science, as Professor Koch has proved himself, he did not hesitate to experiment upon himself with his injected remedy, and make accurate observations of its effect upon the system, in his own case, as well as upon that of others, whom it was designed to benefit, as the following extract shows :

"The symptoms arising from an injection of 0.25 cubic centimeter I have observed after an injection made in my own upper arm. They were briefly as follows :

"Three hours after the injection there came on pain in the limbs, fatigue, inclination to cough, difficulty of breathing, which speedily increased in the last hour, and were unusually violent.

"A chill followed which lasted almost an hour. At the same time there was nausea, vomiting and rise of body temperature to 39.6 degrees centigrade. After twelve hours all these symptoms abated, the temperature fell, and on the next day it was normal. A feeling of fatigue and pain in the limbs continued for a few days, and for exactly the same period of time the site of injection remained slightly painful and red.

"The smallest quantity of the remedy which will affect the healthy human being is about 0.01 cubic centimeter, equal to one cubic centimeter of the one hundreth dilution. As has been proved by numerous experiments when this dose is used, reaction in most people shows itself only by slight pains in the limbs and transient fatigue. A few showed a rise of temperature to about thirty-eight degrees.

"Although the effect of the remedy in equal doses is very different in animals and in human beings of calculated body weight, in some other qualities there is much similarity in the symptoms produced, the most important of these resemblances being the specific action of the remedy on the tuberculous process, which I will not here describe. I will make no further reference to its effects on animals, but I will at once turn to its extraordinary action on tuberculosis in human beings.

"The healthy human being it does not affect at all, or scarcely at all, as we have seen, when 0.01 cubic centimeter is used. The same holds good with regard to patients suffering from diseases other than tuberculosis, as repeated experiments have proved ; but the case is very different when the disease is tuberculosis. A dose of 0.01 cubic centimeter injected subcutaneously into the tuberculosis patient caused a severe general reaction as well as a local one.

"I gave children aged from two to six years, one-tenth of this dose—that is to say, 0.001 cubic centimeter ; very delicate children only 0.0005 cubic centimeter, and obtained powerful but in no way dangerous reaction. The general reaction consists in an attack of fever, which usually begins with rigors and raises the temperature above 39 degrees, often up to 40 degrees, and even 41 degrees, C. This is accompanied by pain in the limbs, coughing, great fatigue and often sickness and vomiting.

"In several cases a slight icteroid discoloration was observed, and occasionally an eruption like measles on the chest and neck. The attack usually begins four to five hours after the injection and lasts from twelve to fifteen hours. Occasionally it begins later and then runs its course with less intensity. The patients are very little affected by the attack, and as soon as it is over feel comparatively well, generally better than before. The local reaction can be best observed in cases in which the tuberculosis affection is visible, for instance, in cases of lupus, changes take place which show the specific anti-tuberculous action of the remedy to a most surprising degree.

"A few hours after an injection into the skin of the back, that is, in a spot far removed from the diseased area on the face or elsewhere, the lupus begins to swell and to redden, and this it does generally before the initial rigor. During the fever the swelling and redness increase and may finally reach a high degree, so that the lupus tissue becomes brownish and necroentic in places where the growth was sharply defined. We sometimes found a much swollen and brownish spot surrounded by a whitish edge almost one centimeter wide, which again was surrounded by a broad band of bright red.

"After the subsidence of the fever the swelling of the lupus tissue gradually decreases and disappears in about two or three days. The lupus spots themselves are then covered by a safe deposit which filters outward and dries in the air. The growth then changes to a crust, which falls off after two or three weeks, and which sometimes after only one injection—leaves a clean red cicatrix behind. Generally, however, several injections are required for the complete removal of the lupus tissue ; but of this, more later on."

As lupus is a species of cancer, it would seem that the new discovery is likely to prove efficacious in this most fearful class of disorders, as well as in cases of consumption and other tuberculosis diseases. Experiments are still being made with the lymph of Professor Koch, which are watched with intense interest the world over.

